# Documenting end-of-life care plans on the ICU: using a digital proforma may improve compliance with nationally agreed standards

**DOI:** 10.1186/cc9941

**Published:** 2011-03-11

**Authors:** S Cantellow, D Harris, S Smith, C Bordeaux, M Thomas

**Affiliations:** 1 Bristol Royal Infirmary, Bristol, UK

## Introduction

The Liverpool Care Pathway (LCP) is the accepted gold standard in the documentation of end-of-life care in the UK. A computerized version of the LCP in the form of a digital proforma (DP) exists as an option on our unit. Some clinicians choose to use free-text (FT) entry citing as this can be more comprehensive. Our study investigates whether usage of the DP is likely to result in a greater degree of compliance with LCP standards than FT alone.

## Methods

All deaths occurring between 1 January 2009 and 30 June 2009 were identified from the record of ITU admissions. Cases of cardiac arrest and brain stem death were excluded. Quality of documentation was scored by a nurse and doctor assessor (for each LCP goal: 0 = nothing entered, 1 = goal partially addressed, 2 = goal fully addressed). The average of the total scores for each case was calculated. Performance of the DP was analysed by comparing average total scores for DP versus FT alone using simple nonparametric descriptors.

## Results

**Figure 1:**
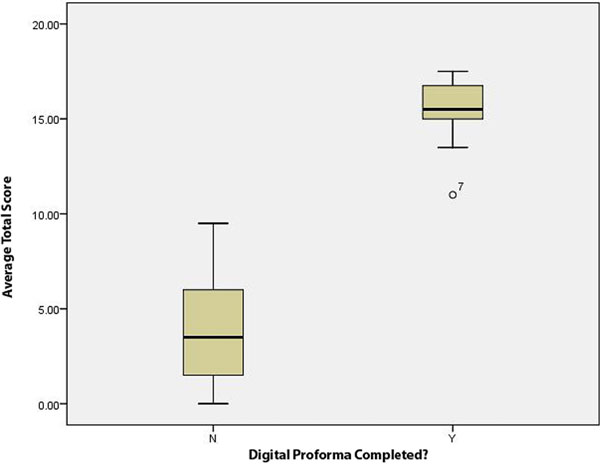


There were 52 deaths and 45 after exclusions. Use of the DP resulted in considerably higher total average scores (range 13.50 to 17.5, interquartile range 15.50 to 16.75, median 15.50) than use of FT alone (range 0.00 to 9.50, interquartile range 1.75 to 6.25, median 4.00). Statistical significance is suggested by the lack of overlap in the range values. See Figure 1.

## Conclusions

Using the DP for end-of-life documentation is highly likely to improve compliance with accepted standards in end-of-life care. Doctors using FT alone were unlikely to document all of the broad issues that require consideration. The use of a DP can function as a useful checklist ensuring patients receive the best care when organ support is withdrawn.

